# Comparative study of three cultivars of jaboticaba berry: nutrient, antioxidant and volatile compounds

**DOI:** 10.3389/fpls.2023.1105373

**Published:** 2023-07-10

**Authors:** Shaosi Xu, Yingying Pang, Xiaoming Cai, Qinchang Chen, Gang Jin, Miao Zhang, Luqiang Huang

**Affiliations:** ^1^ College of Life Science, Fujian Normal University, Fuzhou, China; ^2^ School of Food Science and Engineering, South China University of Technology, Guangzhou, China; ^3^ Institute of Food Inspection, Fujian Institute of Product Quality Supervision and Inspection, National Center for Quality Supervision and Inspection of Processed Foods, Fuzhou, China; ^4^ School of Food and Wine, Ningxia University, Yinchuan, China

**Keywords:** jaboticaba, berry, nutrient, antioxidant, volatile compounds

## Abstract

Jaboticaba is a tropical plant and its fruit rich in nutrients, volatile compounds, and biological activities, which considered to be an edible health benefits plant. Despite its popularity for fresh consumption, jaboticaba is rarely used in intensive processing in China. The content of nutrients and antioxidant in jaboticaba greatly impacts how it is processed healthy food. In this study, we evaluated the nutrients, antioxidant capacity, and volatile compounds of three jaboticaba cultivars including Sabara, Argentina, and Fukuoka, respectively. Our results revealed each variety has its merits. Sabara had an abundance of volatile compounds, a suitable acid-sugar ratio, and a slightly lower antioxidant capacity, making it suitable for fresh consumption. Argentina is the richest in volatile compounds in ripe fruit, but slightly lighter in taste and acid-sugar ratio, making it suitable for dry products. The large size, juicy flesh, low acid-sugar ratio, and less volatile compounds content of Fukuoka also make it suitable for juice processing. Three cultivars of jaboticaba berry exhibited different characteristics, providing reference evidence for the manufacturing and processing of jaboticaba health food.

## 1 Introduction

Jaboticaba (*Myrciaria cauliflora*) belongs to the Myrtle family and is native to South America, which was introduced from Taiwan in 2004 and mostly grow in southern provinces of China (Zhu, 2014; Zhu, 2017). The flower and berry of jaboticaba grow on the main trunk and branches. Due to its berry resemblance to grape, jaboticaba is commonly known as “tree grape”. The jaboticaba skin changes from green to red and then purple as it matures, with only 4 to 7 days of fruiting after ripening. Ripe berry is purple-black, and its flesh is white and semi-translucent crystal (Inada et al., 2021). Jaboticaba tastes sweet and sour, with aromas of guavas, pineapples, mangosteens, and sakas. Qiu (Qiu et al., 2021) measured the volatile components of jaboticaba at different developmental stages *via* headspace-gas chromatography/mass spectrometry and found the most predominant volatile components were monoterpenes, including α-pinene, β-pinene, and D-limonene.

Jaboticaba is also considered as an edible health benefits plant and its pulp is rich in acids, sugars, vitamins, dietary fiber, minerals, and polyphenols (Qiu et al., 2021; Correia et al., 2022; Boldori et al., 2023). There are numerous phenolic substances, including anthocyanins, ellagic acid, and tannins (Pereira et al., 2017; Albuquerque et al., 2020; Fernandes et al., 2020) in the peel, which afford various biological activities, such as antioxidation, anti-inflammatory, antibacterial, hypoglycemic, and lipid-lowering properties (Inada et al., 2018; Zhao et al., 2019; Ferreira et al., 2021; Geraldi et al., 2021; Da Silva-Maia et al., 2023). Through a randomized crossover study, Geraldi (Geraldi et al., 2021) demonstrated jaboticaba juice improved serum antioxidant capacity and plasma glucagon-like peptide-1 response after a carbohydrate meal in healthy adults. Wang (Wang et al., 2014) found that jaboticaba seed extract had anti-proliferative effects on oral cancer cells. Hsu (Hsu et al., 2016) reported an extract from jaboticaba improved diabetic nephropathy by inhibiting oxidative stress and inflammation in streptozotocin-nicotinamide mice. Nayara (Sarkar et al., 2022) have demonstrated that the phenolic compounds and sugars in jaboticaba can be used by colon microbes. Jaboticaba extract promotes the fermentation of intestinal microflora, which is beneficial to human health. As a tropical fruit, the jaboticaba is a potential source of bioactive and functional foods (Massa et al., 2022; Lima et al., 2023).

Many previous studies have proven the nutritional and unique taste of the jaboticaba, but it is the way in which it is processed that has not been well promoted because of its limited freshness and difficulty in storage (Inada et al., 2021). Jaboticaba is very popular throughout Brazil and is consumed as fresh fruit, as well as artisanal products, such as juices, jellies, vinegar, liqueurs, and wines. However, its limited shelf life has hindered the commercialization of fresh fruit. Therefore, to add value and expand its consumption, researchers have been investigating technological processes to develop products derived from jaboticaba (Appelt et al., 2015). The pleasant organoleptic properties and rich nutritional content of jaboticaba make it a functional ingredient in beverage, jams, and jellies (Asquieri et al., 2009; Oliveira et al., 2018; Qiu et al., 2018; Benvenutti et al., 2021). Thus, jaboticaba has high economic benefits due to its consumption as a high-quality natural food ingredient. However, the research on the nutritional composition and active components of the main cultivated varieties of jaboticaba berry is still insufficient. In this study, the immature and ripe fruits of Sabara, Fukuoka, and Argentina were evaluated for their morphological appearance, nutritional composition, antioxidant activity and flavor content. Based on variety of bioactive compounds contained in jaboticaba berry, this study would provide an insight and reference about breeding, preserving, processing of jaboticaba cultivars for both the producer and researcher further research and development.

## 2 Materials and methods

### 2.1 Materials

The unripe and ripe fruits of Sabara, Fukuoka, and Argentina were provided by Fujian Putian Taichuang Agricultural Development Co (Putian Fujian, China). The unripe fruit were collected at 15 days after flowering and the ripe fruit are harvested at 30 days after flowering. 2,2-Diphenyl-1-picrylhydrazyl (DPPH) was purchased from Shanghai Yuanye Biotechnology Co (Shanghai, China). Forinol was purchased from Beijing Solarbio Biotechnology Co (Beijing, China). 2,2’-azino-bis(3-ethylbenzothiazoline-6-sulfonate) (ABTS) was purchased from Sigma-Aldrich Biotechnology Co (St Louis, MO, USA). The total antioxidant capacity (T-AOC) assay kit and hydroxyl radical assay kit were purchased from Nanjing Jiancheng Institute of Biological Engineering (Nanjing Jiangsu, China). Potassium metabisulphite was purchased from LAMOTHE-ABIET, France (Bordeaux, France). Cyclohexanone (chromatographically pure) was purchased from China Pharmaceutical Group Co (Beijing, China). All other chemicals and reagents were analytically pure and purchased from China Pharmaceutical Group Co (Beijing, China).

Three cultivars of jaboticaba were cultivated on a randomly selected plot of land in the farm. During their cultivation, they were given equal amounts of water and organic fertilizer to provide nutrients. Samples were taken before and after the fruit had ripened. In sampling, fruits of the same maturity were taken from several trees of the same cultivar and mixed as samples to be tested. For testing and analysis, three different cultivars of jaboticaba were divided into ripe and unripe groups in order to assess their physicochemical properties and volatile compounds. Around 50 g of randomly selected jaboticaba were placed in 50mL centrifuge tubes to crush them and then placed in an ultrasonic extractor for 30 minutes to leach the substances from the peel and pulp into the juice. Finally, the juice was taken for assay, with three biological replicates.

### 2.2 Methods

#### 2.2.1 Determination of basic chemical parameters

##### 2.2.1.1 Mean fruit diameter and weight

Ten fruits were randomly selected from each of the two periods of the three cultivars. The weight and diameter of each fruit were measured using an electronic balance and vernier calipers, and the mean was calculated.

##### 2.2.1.2 Total soluble solids

Total soluble solids was determined according to the method of NY/T2637-2014 Refractometric method for determination of total soluble solids in fruits and vegetables (The Ministry of Agriculture of the People’s Republic of China, China).

##### 2.2.1.3 Reducing and total sugars

Using the method published by Wu (Wu et al., 2018), with slight modification, reducing sugars in the fruit was determined. A glucose standard curve was generated using 1 mL of glucose standard solutions at concentrations of 0, 0.2, 0.4, 0.6, 0.8, and 1 mg/mL. The modified method was to pipette the solutions into 10 mL colorimetric tubes, and 2 mL 3, 5-dinitrosalicylic acid **(**DNS) reagents (Solarbio, China) were added. The mixture was boiled accurately in a boiling water bath for 5 min and then cooled to 25°C with tap water. Then, 9 mL of distilled water was added to each tube and mixed well. The absorbance values were measured at 540 nm. The final regression equation y = 0.7214x + 0.041 (R² = 0.999) was obtained.

After extracting the reducing sugars, the samples were treated in the same way as described above. The absorbance values were measured, and the reducing sugar content was calculated according to the regression equation.

Total sugars were determined by the phenol-sulfuric acid method, with reference to (Liu et al., 2011).

##### 2.2.1.4 Total acid (as tartaric acid)

Total acid was determined according to the method of “GB 12456-2021 Determination of total acid in foods” (National Health and Family Planning Commission, China).

##### 2.2.1.5 Total polyphenols

Total polyphenols were determined according to the method reported by Li (Li et al., 2020), with slight modification. A standard curve was generated using 1 mL of gallic acid monohydrate standard solutions at concentrations of 0, 10, 20, 30, 40, and 50 µg/mL. The modified method was to pipette the solutions into 10 mL colorimetric tubes, and 5 mL of distilled water, 1 mL of Forinol color developer, and 3 mL of 7.5% sodium carbonate solution were added. The solutions were mixed with a vortex oscillator, and the reaction was carried out at room temperature for 2 h, protected from light. The absorbance was measured at 765 nm, and the regression equation y = 0.0075x - 0.0039 (R^2^ = 0.9996) was obtained. The samples were treated in the same way as described above. The absorbance values were recorded, and the total polyphenols content was calculated according to the regression equation.

##### 2.2.1.6 Vitamins B1 and C

Vitamin C was determined by high-performance liquid chromatography (Waters, USA) according to the method of “GB5009.86-2016 Determination of Ascorbic Acid in Foods” (National Health and Family Planning Commission, China). The determination of vitamin B1 was performed by high-performance liquid chromatography (Waters Corporation, USA), according to the method of “GB 5009.84-2016 Determination of vitamin B1 in foods” (National Health and Family Planning Commission, China).

##### 2.2.1.7 Amino acid content

The amino acids were determined according to the method of “GB5009.127-2016 Determination of amino acids in foods” (National Health and Family Planning Commission, China).

##### 2.2.1.8 Elemental composition

Arsenic, lead, sodium, potassium, magnesium, iron, copper, zinc, calcium, manganese, and mercury were determined using inductively-coupled plasma mass spectrometry (ICP-MS 7900, Agilent, USA), according to the method of “GB5009.268-2016 Determination of Multiple Elements in Foods” (National Health and Family Planning Commission, China). Chromium and cadmium were determined according to the method of “GB5009.123-2014 Determination of Chromium in Foods” and “GB5009.15-2014Determination of Cadmium in Foods” (National Health and Family Planning Commission, China). The determination of cadmium in food was carried out using an atomic absorption spectrophotometer (PE AA 900T, PE Inc., USA). Mercury was determined *via* an atomic fluorescence photometer, according to the method of “GB5009.17-2021 Determination of Mercury in Foods” (National Health and Family Planning Commission, China) using an Atomic Fluorescence Photometer (AFS-930, Beijing Jitian Instruments Co., Ltd., China).

#### 2.2.2 *In vitro* antioxidant assay

##### 2.2.2.1 Total antioxidant capacity

A total antioxidant capacity (T-AOC) assay kit (A015-1-2, Nanjing Jiancheng Institute of Biological Engineering, China) was employed for the determination of antioxidant activity.

##### 2.2.2.2 Hydroxyl radical inhibition

A hydroxyl free radical assay kit (A018-1-1, Nanjing Jiancheng Institute of Biological Engineering, China) was used to determine the hydroxyl radical inhibition.

##### 2.2.2.3 DPPH scavenging capacity

The method of Liu (Liu et al., 2022) was referenced and modified.

The DPPH standard was accurately weighed to 7.8864 mg and fixed to 100 mL with anhydrous ethanol was added to obtain an ethanolic solution of DPPH at a concentration of 0.2 mmol/L. The solution was transferred to a brown bottle and set aside. The sample was added to the test tube according to **Table 1** and mixed well. The absorbance was measured at 517 nm after 30 min reaction at room temperature, protected from light.

**Table 1:** The amount of DPPH added.

Absorbance value	Amount of sample added
Ao	1mL DPPH ethanol solution + 2mL anhydrous ethanol + 1mL blank solvent
As	1mL DPPH ethanol solution + 2mL anhydrous ethanol + 1mL sample solution
Aj	3mL anhydrous ethanol + 1mL sample solution

DPPH scavenging was calculated according to the following formula:


(1) $$Removal\;rate(\%)=\left[1-\frac{As-Aj}{Ao}\right]\times 100\%$$

##### 2.2.2.4 Determination of the ability to scavenge ABTS cationic radicals

The ABTS assay was performed according to the procedures described by Tao (Tao et al., 2016; Wang et al., 2022), with some modifications. Briefly, 96 mg of ABTS was dissolved in 20 mL of deionized water, and then 5 mL of 2.45 mmol/L potassium persulfate solution was added. The mixture was left overnight (12–16 h) in the dark at room temperature before use. The obtained ABTS^+^ radical solution was diluted 11-fold with ethanol, to achieve an absorbance of 0.70 (± 0.02) at 734 nm. In the next step, 20 µL of juice (with proper dilution in ethanol) was added to 2 mL of the aforementioned ABTS^+^ solution. In addition, 20 µL of ethanol was added to 2 mL of the ABTS+ solution, and this sample was the blank. After incubation at 30°C in the dark for 6 min, the absorbance at 734 nm was recorded. The results are expressed as the percent ABTS^+^ radical inhibition.

The scavenging capacity was calculated as follows:


(2) $$Inhibition\%=(1-\frac{Asample}{Ablank})\times 100$$

##### 2.2.2.5 Inhibit superoxide anion radicals

An inhibition and generation of superoxide anion radical assay kit (A052-1-1, Nanjing Jiancheng Institute of Biological Engineering, China) was used to determine the inhibition of superoxide.

#### 2.2.3 Volatile compounds

The volatile compounds were determined on an HS-SPME/GC–MS system (Agilent, California, USA) (Ricci et al., 2019). Briefly, 5 mL of samples and 3.6 g of NaCl were added to the headspace bottle and equilibrated at 50°C for 5 min. The extraction fiber was inserted into the sample bottle for 30 min. The oven temperature program was as follows: hold at 40°C for 3 min, increase to 60°C at a rate of 2°C/min and hold for 5 min, increase to 150°C at a rate of 3°C/min and hold for 10 min, increase to 220°C at a rate of 5°C/min and hold for 10 min. Volatile compounds were identified by comparison of the retention time and mass spectra with pure standards in the NIST 17 mass spectral and retention index libraries. Quantitative analysis was performed using cyclohexanone purchased from China Pharmaceutical Group (Beijing, China) as the internal standard.

### 2.3 Statistical analysis

Statistical analysis was performed using Statistical Product Service Solutions (SPSS), version 20.0 (IBM Corp., Armonk, NY, USA) and Prism, version 8.0 (GraphPad Software Inc., San Diego, CA, USA). The data are expressed as the mean ± SD of three biological replicates. The one-way analysis of variance (ANOVA) within the ripe and unripe groups followed by Tukey’s test and *P*< 0.05 was considered statistically significant. PCA analysis using the R package prcomp. All analysis were visualized using Prism, version 8.0.

## 3 Results and discussion

By examining the differences in quality between three different cultivars of jaboticaba, we identified suitable cultivars for fresh consumption and processing. To investigate the quality of fruits upon maturity, we examined various quality parameters of the fruit at unripe and ripe stages. The three varieties of jaboticaba fruits had excellent quality at the unripe stage, and differences were found upon ripening. Generally, we consume only the ripe fruits; thus, in the following discussion and analysis, we focus on analyzing the differences of these three kinds of Jaboticaba fruits in the ripening stage, using the unripe fruits as controls.

### 3.1 Fundamental properties and chemical analysis

**Figure 1:**
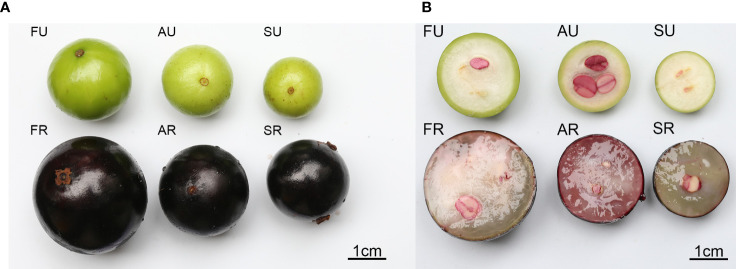
Three cultivars of jaboticaba berry. Appearance of a jaboticaba **(A)**; cross-sectional section of a jaboticaba **(B)**. SU represent Sabara unripe, AU, Argentina unripe; FU, Fukuoka unripe; SR, Sabara ripe; AR, Argentina ripe; FR, Fukuoka ripe.

The appearance of the three different cultivars of jaboticaba is shown in **Figure 1A**, and the cross-sectional cut is shown in **Figure 1B**. From this figure we can see that the outer skin of the jaboticaba fruit is black at the ripening stage and green when unripe. Superficially, the jabotic fruits of the three cultivars have large size differences. In cross-section, the three cultivars also differ considerably in flesh color and form and seeds.

In addition, the fundamental properties and chemical composition of unripe and ripe jaboticaba fruit of three different cultivars were determined. The average diameter and weight were measured to determine the size of the fruit. The contents of amino acids, vitamin C, vitamin B1, reducing sugar, total sugar, total acid, and total polyphenols were measured to determine their nutritional and volatile compounds.

#### 3.1.1 The average diameter and weight

The average size of the fruit is summarized in **Table 2**. The average diameter of the three different fruits, both in the unripe and ripe stages, was larger in Fukuoka than in Argentina and Sabara. At maturity, the average diameter of Fukuoka reached 31.10 ± 2.71 mm; Argentina was 25.61 ± 2.05 mm; Sabara was only 24.12 ± 1.17 mm. The diameter of the ripe fruit of Fukuoka was significantly different from that of Sabara and Argentina, but at the unripe stage, Sabara was significantly different from the other two cultivars. Similar to the average diameter, the average weight followed the same trend. In the immature stage, Fukuoka and Argentina were significantly heavier than Sabara, and in the mature stage, Fukuoka was significantly heavier than Argentina and Sabara. In the mature stage, Fukuoka reached an average weight of 16.76 ± 3.63 g, while Argentina and Sabara were only 9.93 ± 2.54 g and 8.26 ± 0.7 g, respectively.

**Table 2:** Average weight and diameter of jaboticaba at unripe and ripe stages.

Type	Ripe fruit			Unripe fruit		
	Sabara	Argentina	Fukuoka	Sabara	Argentina	Fukuoka
Average diameter(mm)	24.12 ± 1.17b	25.61 ± 2.05b	31.10 ± 2.71a	20.64 ± 1.30b	23.74 ± 3.07a	24.41 ± 1.98a
Average weight (g)	8.26 ± 0.7b	9.93 ± 2.54b	16.76 ± 3.63a	5.39 ± 0.77b	7.49 ± 2.45a	8.45 ± 1.78a

Different letters indicated significant differences within the ripe and unripe groups. *p*<0.05.

For each of the three jaboticaba cultivars, we measured the average weight and diameter and found that Fukuoka was the largest, followed by Argentina and Sabara. This suggests that Fukuoka is better than Argentina and Sabara, in terms of fruit size. From a consumer’s point of view, when buying fruit most consumers will pick the larger ones, so in terms of weight per fruit, the Fukuoka is probably more popular for fresh eating.

#### 3.1.2 Total soluble solids, total sugar, reducing sugar and acidity

**Figure 2:**
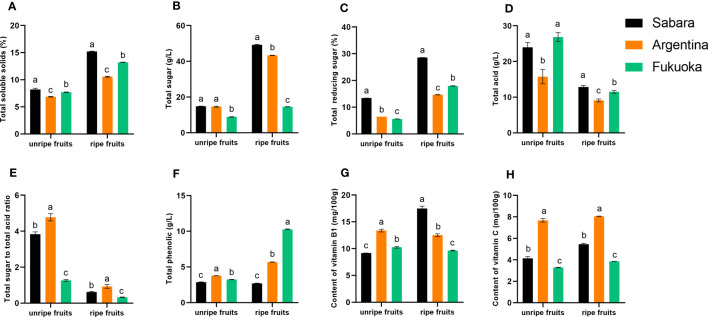
Physical and chemical characteristics of jaboticaba. **(A–H)** represent the total soluble solids, total sugar, total reducing sugar, total acid, total sugar to total acid ratio, total polyphenols, Vitamin B1 content, and Vitamin C content of different jaboticaba, respectively. Different lowercases indicate significant difference within the ripe and unripe groups. *p*<0.05.

In general, both for unripe and ripe fruit, total soluble solids content was highest in Sabara, followed by Fukuoka and Argentina (**Figure 2A**). In the ripe fruit, total soluble solids content reached 15.22% in Sabara and 13.23% in Fukuoka, while in Argentina it was only 10.56%. Among the unripe fruits, total soluble solids content reached 8.21% in Sabara and 7.7% in Fukuoka, while in Argentina it was only 6.87%. Notably, Sabara exhibited significantly higher total soluble solids content than the other two cultivars.

In general, both for unripe and ripe fruit, the total sugar content was highest in Sabara, followed by Argentina and Fukuoka (**Figure 2B**). In the ripe fruit, the total sugar content reached 49.22 g/L in Sabara and 43.30 g/L in Argentina, while in Fukuoka it was only 14.54 g/L. The total sugar content of Sabara and Argentina was almost three folds that of Fukuoka. To better evaluate of the sugar content of these jaboticaba cultivars, we also determined the percentage of reducing sugar in the total sugar. The trend in the percentage of reducing sugar differed between ripe and unripe fruit (**Figure 2C**). Among the unripe fruits, Sabara had the highest percentage of reducing sugar, followed by Argentina and Fukuoka. Among the ripe fruits, the highest percentage of reducing sugar was found in Sabara, followed by Fukuoka and then Argentina. Notably, Sabara exhibited significantly higher total sugar content and reducing sugar percentage than the other two cultivars.

In terms of total acidity, unripe and ripe fruit have different trends (**Figure 2D**). The acidity of unripe fruit was highest in Fukuoka, followed by Sabara and Argentina. On the contrary, Sabara was more acidic than Fukuoka and Argentina, in the ripe fruit. In addition, the acidity decreased as the fruit matured, and ultimately, the acidity of the three fruits was not significantly different at approximately 10 g/L. The ratio of total sugar to total acidity is presented in **Figure 2E**.

The content and composition of acids and sugars are important quality factors that directly influence the flavor and acceptability of fruits and berries (Tang et al., 2001; Liu et al., 2010). Fruit acids promote food digestion and improve blood circulation. The acid content may also affect the stability of phenolic compounds in the fruit (Chang et al., 2006). We determined the sugar and acid concentrations separately and calculated the sugar-acid ratio. There were significant differences in the sugar-acid ratios of the three jaboticaba cultivars. In general, a high sugar-to-acid ratio means the fruit has a sweet and sour taste. From this, it can be concluded that Argentina and Sabara had more desirable flavor. Since Fukuoka had a lower sugar-to-acid ratio, it is better suited for use in processed products.

#### 3.1.3 Total polyphenols

The total polyphenols content of the ripe fruit was higher than in unripe for all cultivars except Sabara (**Figure 2F**). For the unripe fruit, the difference in total polyphenols content between the three cultivars of jaboticaba was not significant, all at around 3 g/L. However, when the fruit was ripe, there were very significant differences in total polyphenols content between the cultivars. In Sabara, the total polyphenols content was only 2.69 g/L. Argentina had 5.68 g/L, and the highest was Fukuoka with 10.27 g/L, about three times that of Sabara and twice that of Argentina.

Jaboticaba fruit is a dark berry rich in phenolic compounds, particularly anthocyanins and ellagitannins (Wu et al., 2012; Inada et al., 2015; Plaza et al., 2016; Pereira et al., 2017). The phenolic content has been studied by a large number of scholars who discovered many potential uses for jaboticaba (Lee et al., 2017; Rodrigues et al., 2021; Soares et al., 2021). In this study, we obtained results that are consistent with those of previous researchers. We also found large differences between the three jaboticaba cultivars. In particular, the total polyphenols content of Fukuoka was extremely high. Phenolic compounds derived from jaboticaba could be used to treat inflammation. In South American countries, such as Brazil, people take jaboticaba directly to combat some inflammation (Brito et al., 2021). Therefore, the consumption of Fukuoka could provide good anti-inflammatory effects, and intensive processing of Fukuoka is likely.

#### 3.1.4 Vitamin B1 and Vitamin C

The vitamin content varied slightly between the three cultivars of jaboticaba (**Figures 2G, H**). The vitamin B1 content in Sabara increased with fruit maturity, whereas the opposite was true for Argentine and Fukuoka. Moreover, the ripe Sabara fruit had a much higher vitamin B1 content than Argentina and Fukuoka, reaching 17.47 mg/100 g. The trend for vitamin C differed from vitamin B1. The vitamin C content was highest in Argentina, followed by Sabara and Fukuoka for unripe and ripe fruits, and the vitamin C content increased with fruit maturity. Argentina had 8.06 mg/100 g of vitamin C in ripe fruit, compared to 3.86 mg/100 g in Fukuoka, which is about two times higher. In general, jaboticaba has a slightly higher vitamin C content than grapes (Tavasolinasab et al., 2023).

Vitamin B1 has been reported to be effective in reducing the risk of depression (Nguyen et al., 2022), and vitamin C has good antiviral (Colunga Biancatelli et al., 2020) and antioxidant properties, as well as potential benefits for skin health (Pullar et al., 2017). Previously, it was verified that jaboticaba contains a wide range of vitamins. From our results, Argentina had a much higher vitamin C content than Sabara and Fukuoka, but Sabara has a much higher vitamin B1 content than Argentina and Fukuoka. It can therefore be concluded that in terms of vitamins, Sabara and Argentina are of higher quality than Fukuoka. However, vitamins can easily lose their activity at high temperatures or under other conditions. In this respect, Argentina and Sabara should be consumed fresh to maximize their vitamin absorption by the human body.

#### 3.1.5 Amino acids

Jaboticaba has been reported to be rich in amino acids, and our findings are consistent with these reports. In our study, we detected 16 amino acids, such as aspartic acid, glutamic acid, and threonine, in different cultivars of jaboticaba, and the detailed data are shown in **Table 3**. Both unripe and ripe fruits contained these 16 amino acids, with differences in their content. In the case of Sabara, the most abundant amino acid was glutamic acid at 0.69 g/kg, and the least abundant was methionine at 0.03 g/kg in unripe fruit. At maturity, the amino acid with the highest concentration was glutamic acid at 0.66 g/kg, and the lowest was methionine at 0.04 g/kg. For Argentina, the most abundant amino acid in ripe fruit was glutamic acid at 0.64 g/kg and the least abundant was methionine at 0.03 g/kg. At unmaturity, the most abundant amino acid was leucine at 0.39 g/kg, and the least abundant was methionine at 0.02 g/kg. For Fukuoka, the most abundant amino acid was glutamic acid at 0.63 g/kg and the least abundant was methionine at 0.03 g/kg in unripe fruit. At maturity, the amino acid with the highest concentration was glutamic acid at 0.75 g/kg, and the lowest was methionine at 0.03 g/kg.

**Table 3:** Concentration of amino acid (g/Kg) in jaboticaba at unripe and ripe stages.

Amino acid	Ripe fruit			Unripe fruit		
	Sabara	Argentina	Fukuoka	Sabara	Argentina	Fukuoka
Asp	0.50 ± 0.00c	0.36 ± 0.01a	0.46 ± 0.00b	0.60 ± 0.01b	0.51 ± 0.01c	0.53 ± 0.00a
Thr	0.22 ± 0.00b	0.24 ± 0.00b	0.21 ± 0.00c	0.27 ± 0.00c	0.25 ± 0.00a	0.24 ± 0.00a
Ser	0.27 ± 0.00b	0.11 ± 0.00a	0.23 ± 0.00b	0.30 ± 0.00c	0.29 ± 0.00c	0.26 ± 0.00a
Glu	0.66 ± 0.00b	0.29 ± 0.00b	0.75 ± 0.00a	0.69 ± 0.01b	0.64 ± 0.01c	0.63 ± 0.00a
Gly	0.27 ± 0.01c	0.31 ± 0.00b	0.25 ± 0.00c	0.29 ± 0.00b	0.25 ± 0.00a	0.26 ± 0.00a
Ala	0.38 ± 0.00b	0.25 ± 0.00a	0.31 ± 0.00b	0.36 ± 0.00c	0.34 ± 0.01c	0.29 ± 0.00a
Val	0.23 ± 0.00b	0.25 ± 0.00b	0.22 ± 0.00c	0.29 ± 0.00b	0.26 ± 0.00a	0.26 ± 0.00a
Met	0.04 ± 0.00a	0.02 ± 0.00a	0.03 ± 0.00b	0.03 ± 0.00b	0.03 ± 0.00c	0.03 ± 0.00a
Ile	0.17 ± 0.01b	0.03 ± 0.00a	0.16 ± 0.00a	0.22 ± 0.00b	0.20 ± 0.00b	0.20 ± 0.00a
Leu	0.32 ± 0.01b	0.39 ± 0.00b	0.31 ± 0.00b	0.41 ± 0.01b	0.38 ± 0.01a	0.38 ± 0.00a
Tyr	0.09 ± 0.00b	0.05 ± 0.00b	0.11 ± 0.00a	0.17 ± 0.00b	0.16 ± 0.00c	0.15 ± 0.00a
Phe	0.22 ± 0.00b	0.19 ± 0.00a	0.21 ± 0.00b	0.27 ± 0.00b	0.25 ± 0.00c	0.25 ± 0.00a
Lys	0.35 ± 0.01b	0.25 ± 0.00a	0.34 ± 0.01b	0.46 ± 0.00c	0.43 ± 0.01c	0.39 ± 0.00a
His	0.16 ± 0.01b	0.12 ± 0.00a	0.13 ± 0.00b	0.21 ± 0.00c	0.19 ± 0.00c	0.16 ± 0.00a
Arg	0.23 ± 0.00b	0.17 ± 0.00a	0.23 ± 0.00a	0.31 ± 0.00c	0.29 ± 0.00b	0.26 ± 0.00a
Pro	0.25 ± 0.00b	0.11 ± 0.00a	0.23 ± 0.01b	0.27 ± 0.00b	0.24 ± 0.00c	0.24 ± 0.01a

Different letters indicated significant differences within the ripe and unripe groups. *p*<0.05.

Amino acids are essential nutrients, but they cannot be synthesized by the body. They must be replenished through the consumption of foods rich in amino acids (Kurpad et al., 2006). In this work, all three varieties of jaboticaba were rich in a variety of amino acids, and the amino acid content was slightly different between cultivars. Amino acids, such as glutamic acid, arginine, aspartic acid, and cysteine, have biological activities in the treatment of several diseases and have been used in clinical applications (Ferrando et al., 2005; MacDonald et al., 2019). Our results demonstrate the contents of aspartic acid, glutamic acid, and glycine were relatively high in jaboticaba fruits, in comparison to other amino acids. In addition, for the 16 amino acids detected, Argentina generally had lower levels than Fukuoka and Sabara, with the lysine, leucine, etc. We can assume that Fukuoka and Sabara contain more amino acids than Argentina, making them excellent choices in a healthy diet.

#### 3.1.6 Elemental analysis

Trace elements are important to human wellness; thus, we examined the trace element content of the three jaboticaba cultivars, and the data are summarized in **Table 4**. Significant differences in the content of each element were found. K, Ca and Mg were present in significant amounts, and Cu, Al, and Sr were present at low levels relative to the other elements. Toxic elements including As, Cr, and Cd were detected at remarkably low and almost negligible levels. Of the 25 vital elements closely associated with the human body, essential macro elements, including K, Mg, and Ca, and essential microelements, such as Zn, Fe, Cu, and Mn, play an extremely important role in the control of human diseases (Anuk et al., 2021; Budinger et al., 2021; Zeidan et al., 2021). We also found some difference in trace element content between unripe and ripe fruit, with these elements except Al, Mn, Cu, As, Sr, and Ba were slightly higher in ripe fruit than in unripe fruit. The results indicate the highest trace element content in Sabara, Argentina, and Fukuoka was K, followed by Mg. Furthermore, Argentina had slightly higher trace element content than Sabara and Fukuoka, which would suggest that Argentina contains the most beneficial trace elements. It is also worth noting that all three cultivars of jaboticaba contain Fe and Zn, which are also beneficial to human wellness.

**Table 4:** Concentration of elemental (mg/Kg) in jaboticaba at unripe and ripe stages.

Element	Ripe fruit			Unripe fruit		
	Sabara	Argentina	Fukuoka	Sabara	Argentina	Fukuoka
Na	3.00 ± 0.00a	3.00 ± 0.00a	3.00 ± 0.00a	3.00 ± 0.00a	3.00 ± 0.00a	3.00 ± 0.00a
Mg	119.06 ± 3.08b	116.97 ± 1.44a	106.67 ± 2.97b	110.61 ± 1.16c	107.73 ± 0.53a	99.66 ± 1.86b
Al	0.97 ± 0.06a	0.74 ± 0.31a	0.89 ± 0.17a	0.93 ± 0.32a	1.27 ± 0.29a	0.95 ± 0.13a
K	1746.16 ± 39.10b	1639.45 ± 8.69a	1415.36 ± 25.82c	1274.53 ± 12.44b	1528.64 ± 6.97a	1368.09 ± 33.00c
Ca	78.44 ± 0.26b	68.48 ± 3.17a	56.24 ± 3.30c	123.65 ± 1.46b	67.03 ± 1.13c	94.36 ± 6.09a
Ti	ND	ND	ND	ND	ND	ND
V	ND	ND	ND	ND	ND	ND
Cr	ND	ND	ND	ND	ND	ND
Mn	3.03 ± 0.04a	4.20 ± 0.04a	3.98 ± 0.06b	7.35 ± 0.17c	6.39 ± 0.03b	3.00 ± 0.07a
Fe	2.21 ± 0.07b	1.52 ± 0.05b	1.32 ± 0.02b	1.27 ± 0.18b	1.41 ± 0.06a	1.19 ± 0.06ab
Co	ND	ND	0.02 ± 0.00a	0.02 ± 0.00b	ND	0.00 ± 0.00a
Ni	ND	ND	ND	ND	ND	ND
Cu	0.37 ± 0.01b	0.23 ± 0.02a	0.88 ± 0.01a	0.95 ± 0.03a	0.64 ± 0.02b	0.89 ± 0.03a
Zn	3.85 ± 0.03a	3.12 ± 0.62a	2.79 ± 0.35b	4.49 ± 0.30a	3.41 ± 0.11a	3.99 ± 0.74a
As	0.01 ± 0.00b	0.01 ± 0.00b	0.01 ± 0.00b	0.02 ± 0.00a	0.01 ± 0.00c	0.02 ± 0.00b
Sr	0.13 ± 0.00c	0.14 ± 0.00a	0.29 ± 0.01a	0.53 ± 0.02b	0.12 ± 0.00c	0.26 ± 0.00a
Cd	ND	ND	ND	ND	ND	ND
Sn	ND	ND	ND	ND	ND	ND
Sb	ND	ND	ND	ND	ND	ND
Ba	0.55 ± 0.10b	0.37 ± 0.05a	0.61 ± 0.01a	0.95 ± 0.01b	0.29 ± 0.08b	0.42 ± 0.03a
Hg	ND	ND	ND	ND	ND	ND
Pb	ND	ND	ND	ND	ND	ND

Different letters indicated significant differences within the ripe and unripe groups. ND indicates not detected. *p*<0.05.

Similar to the amino acid content, trace elements must be supplemented through the external intake. In particular, trace elements, such as calcium, iron, and potassium, are beneficial and necessary to human wellness, but some elements, such as lead and mercury, are harmful (Zeidan et al., 2021; Nag and Cummins, 2022; Pierezan et al., 2022). In this study, all three cultivars of jaboticaba had some amount of beneficial trace elements, and no harmful elements were detected. This result further confirmed the consumption of jaboticaba as a nutritional food. In addition, our results showed the overall micronutrient content of Sabara was slightly higher than the other two cultivars, which means that Sabara may be more preferred by consumers.

### 3.2 Analysis of the capacity of antioxidants *in vitro*

The content of phenolic compounds is closely related to antioxidant capacity (Chang et al., 2019). Therefore, to better investigate the best way to consume these three cultivars of jaboticaba, we tested several *in vitro* antioxidant indicators.

#### 3.2.1 Total antioxidant capacity

**Figure 3:**
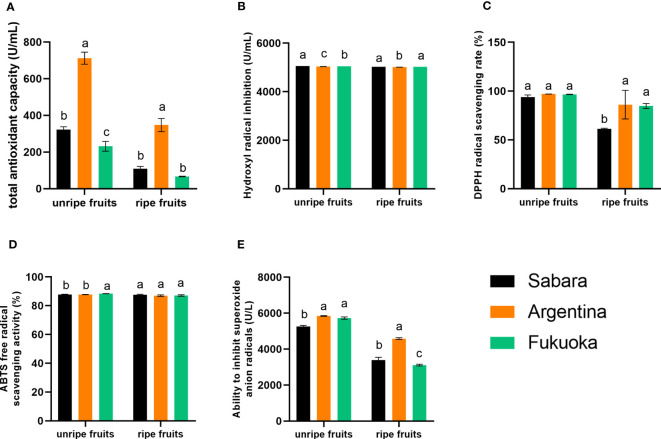
Antioxidant capacity of jaboticaba. **(A–E)** represent the antioxidant capacity, hydroxyl radical inhibition capacity, DPPH radical scavenging rate, ABTS free radical scavenging activity, and ability to inhibition superoxide anion radicals of different jaboticaba, respectively. Different lowercases indicate significant difference within the ripe and unripe groups. *p*<0.05.

The results of the total antioxidant capacity test are shown in **Figure 3A**. The trend was the same for both unripe and ripe fruit, with Argentina having a higher total antioxidant capacity than Sabara and Fukuoka. Additionally, the unripe fruit had a higher total antioxidant capacity than the ripe fruit of the corresponding cultivars. The total antioxidant capacity of the three cultivars of jaboticaba differed significantly, with the highest total antioxidant capacity in Argentina being almost twice that of Sabara and three times that of Fukuoka.

#### 3.2.2 Hydroxyl radical inhibition

The ability to the hydroxyl radical inhibiting capacity of jaboticaba was similar (**Figure 3B**), either between cultivars or between different stages of ripening. The hydroxyl radical inhibitory capacity of Sabara was 5023.23 U/mL in ripe fruit and 5055.78 U/mL in unripe fruit, while that of Argentina was 5010.48 U/mL in ripe fruit and 5034.84 U/mL in unripe fruit. Therefore, we conclude that the hydroxyl radical inhibiting capacity of unripe fruit was slightly higher than that of ripe fruit, and the inhibiting capacity of Sabara was higher than that of Fukuoka and Argentina.

#### 3.2.3 DPPH radical scavenging rate

The results of the DPPH radical scavenging rate are shown in **Figure 3C**. There was no significant difference in the DPPH radical scavenging rate between the three cultivars of unripe jaboticaba, and all samples reached more than 93.75%. The highest scavenging rate of 97.03% for DPPH radicals was found in Argentina. However, in the ripe fruit, all three cultivars of jaboticaba showed a decrease in DPPH radical scavenging, compared to the unripe fruit. In particular, Sabara fruit exhibited a significant decrease from 93.75% to 61.34%. The other two cultivars also decreased but both still had more than 80% DPPH scavenging, with 86.16% in Argentina and 84.77% in Fukuoka. In general, all three cultivars exhibited good scavenging ability for DPPH free radicals.

#### 3.2.4 ABTS free radical scavenging activity

There were no significant differences in the ABTS free radical scavenging capacity of jaboticaba cultivars, either between unripe and ripe fruit or between cultivars (**Figure 3D**). Among the unripe fruits, the strongest scavenging ability was that of Fukuoka, at 88.38%, and the lowest scavenging ability was that of Argentina, at 87.80%. Among the ripe fruits, the strongest scavenging ability was found in Sabara, at 87.64%, and the lowest scavenging ability was also found in Argentina, at 86.99%. Thus, the best scavenger of ABTS free radicals was Sabara, followed by Fukuoka and Argentina; however, the differences between their scavenging abilities were very small (i.e., the values were very close).

#### 3.2.5 Ability to inhibit superoxide anion radicals

The ability of the fruit to inhibit superoxide anion radicals is shown in **Figure 3E**. There were differences in the results, both between cultivars and between ripening periods. The superoxide anion radical inhibition capacity of unripe fruit was higher than that of ripe fruit, and there was a relatively large difference. The superoxide anion radical inhibition capacity of ripe Sabara fruit was 3387.26 U/L, while that of unripe fruit reached 5263.81 U/L. The superoxide anion radical inhibition capacity of ripe Argentina fruit was 4584.87 U/L, while that of unripe fruit reached 5843.70 U/L. The superoxide anion radical inhibition capacity of ripe Fukuoka fruit was 4584.87 U/L, while that of unripe fruit reached 5843.70 U/L. In summary, the superoxide anion radical inhibiting capacity of Argentina was higher than that of Sabara and Fukuoka.

Interestingly, not all of the *in vitro* antioxidants we tested were higher in one variety than in the other two, but each had its own advantages. For example, Argentina had the highest total antioxidant capacity, superoxide anion inhibition capacity, and DPPH scavenging rate among the three. Sabara had the highest hydroxyl radical inhibition capacity and ABTS cation scavenging rate among the three. Although Fukuoka was not the highest among several indexes we tested, it still presented a high antioxidant capacity. Thus, the antioxidant capacity of all three cultivars of jaboticaba was similar, and all were considered to have strong antioxidant capacity. Combining the total polyphenols content and the *in vitro* antioxidant assay results, we believe that Argentina and Fukuoka have stronger anti-inflammatory and antioxidant properties than Sabara. In general, we believe that those with weaker antioxidant properties are not suitable for processed products and are more suitable for fresh consumption. Therefore, we concluded Argentine and Fukuoka are more suitable for processed products, and Sabara is more suitable for fresh consumption. The high polyphenol content of the three cultivars and their strength *in vitro* antioxidant capacity could contribute to anti-aging in humans. This means that all three cultivars of kapok fruit have the perspective of being able to become a medicinal food source.

### 3.3 Volatile compounds analysis

**Figure 4:**
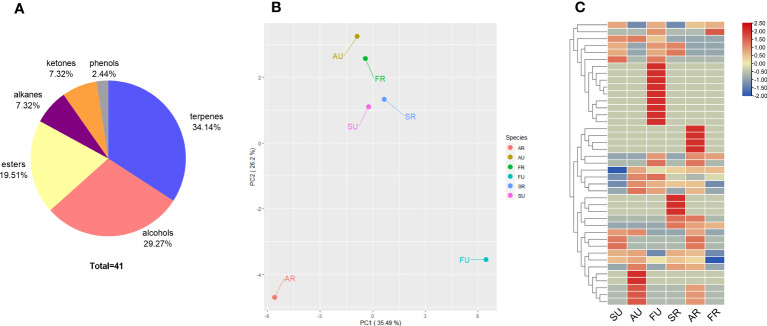
The volatile compounds of jaboticaba. Classification and proportion of total 41 volatile compounds detected in jaboticaba **(A)**; PCA among samples of jaboticaba at different stages and periods **(B)**; heat map displays the hierarchical clustering of volatile compound content in ug/L in the difference within the ripe and unripe groups, with red indicating high levels of content and blue indicating low levels. SU represent Sabara unripe, AU, Argentina unripe; FU, Fukuoka unripe; SR, Sabara ripe; AR, Argentina ripe; FR, Fukuoka ripe **(C)**.

The data from the analysis of the volatile compounds are summarized in **Table 5**. The volatile compounds detected in the three fruit cultivars were mainly alkenes and esters. In addition, for ripe fruit, a greater variety of aromatic substances was detected in Argentina and Sabara. And varieties of volatile compounds were changed as the fruit matured. In contrast, Fukuoka had 22 volatile compounds in unripe fruit but only six in ripe fruit. A total of 41 volatile compounds were identified in Sabara, Argentina, and Fukuoka by HS-SPME-GC/MS. The volatile compounds were divided into terpenes, alcohols, esters, alkanes, ketones, and phenols (**Figure 4A**). Among them, terpenes contributed the most (approximately 34%), followed by alcohols (29%), esters (20%), alkanes (7%) and ketones (7%).

**Table 5:** Concentration of volatile compounds (μg/L) in jaboticaba at unripe and ripe stages.

Volatile compounds	Ripe fruit			Unripe fruit		
	Sabara	Argentina	Fukuoka	Sabara	Argentina	Fukuoka
Terpenes:						
Copaene	288.83 ± 221.3	160.82 ± 19.73	ND	ND	867.71 ± 82.87	ND
Levo-b-elemene	273.32 ± 34.3	321.38 ± 80.81	ND	ND	2161.03 ± 171.95	596.11 ± 467.39
Caryophyllene	542.21 ± 22.48	378.73 ± 45.98	663.22 ± 205.09	186.51 ± 158.95	3127.14 ± 1.68	4195.14 ± 93.31
γ-Muurolene	ND	174.72 ± 74.2	ND	ND	1749.31 ± 996.64	ND
Viridiflorene	ND	205.81 ± 24.42	ND	ND	2813.02 ± 1740.41	494.61 ± 47.85
Germacrene D	ND	257.8 ± 45.4	ND	359.69 ± 408.56	637.07 ± 76.99	ND
(-)-α-Muurolene	ND	ND	ND	ND	1672.77 ± 948.53	ND
Alloaromadendrene	ND	243.05 ± 77.87	ND	ND	ND	ND
(+)-α.-Muurolene	ND	119.88 ± 6.25	ND	160.77 ± 133.18	ND	ND
Aromandendrene	ND	ND	ND	ND	ND	387.85 ± 123.01
(+)-b-Selinene	ND	ND	ND	ND	ND	201.56 ± 6.65
D-Limonene	397.14 ± 248.69	ND	ND	ND	ND	ND
d-Cadinene	ND	175.03 ± 43.18	ND	ND	1677.47 ± 250.47	ND
Bicyclogermacren	ND	336.17 ± 64.79	ND	ND	ND	ND
Alcohols:						
Himbaccol	ND	ND	ND	ND	344.3 ± 114.27	ND
α.-Cadinol	ND	ND	145.87 ± 35.58	ND	206.1 ± 148.46	27.51 ± 19.61
Linalool	581.9 ± 47.14	ND	ND	ND	ND	ND
α.-Terpineol	ND	68.31 ± 4.51	ND	55.42 ± 2.61	ND	ND
Phenylethyl Alcohol	ND	99.25 ± 24.99	172.88 ± 56.78	223.49 ± 88.93	ND	193.96 ± 37.13
Espatulenol	ND	82.65 ± 15.96	ND	ND	ND	89.96 ± 32.99
Eucalyptol	471 ± 153.22	ND	ND	ND	ND	273.21 ± 44.07
Ledol	ND	ND	ND	ND	ND	44.72 ± 15.27
2-((3R,3aR,3bS,4R,7R,7aS)-3,7-Dimethyloctahydro-1H-cyclopenta[1,3]cyclopropa[1,2]benzen-4-yl)propan-2-ol	ND	ND	ND	ND	ND	74.44 ± 20.69
(-)-Globulol	ND	ND	ND	ND	ND	48.29 ± 15.77
Rosifoliol	ND	ND	ND	ND	ND	28.72 ± 9.72
3-Hexen-1-ol, (E)-	125.63 ± 38.2	ND	ND	ND	ND	ND
Esters:						
Benzoic acid methyl ester	1438.35 ± 65.47	309.89 ± 125.67	168.09 ± 62.59	132.72 ± 22.22	ND	ND
Decanoic acid ethyl ester	ND	255.78 ± 70.04	154.67 ± 40.84	ND	ND	185.02 ± 88.74
Octanoic acid ethyl ester	ND	ND	180.48 ± 60.78	ND	ND	42.13 ± 10.38
Methyl trans-cinnamate	298.27 ± 34.64	ND	ND	ND	ND	ND
Benzoic acid ethyl ester	756.82 ± 265.17	ND	ND	144.01 ± 15.55	ND	147.39 ± 64.32
Butanedioic acid diethyl ester	ND	ND	ND	ND	ND	77.55 ± 6.36
(Z)-Ethyl cinnamate	ND	ND	ND	ND	ND	173.62 ± 12.86
Diethyl Phthalate	93.76 ± 4.41	145.81 ± 25.19	ND	124.32 ± 15.44	231.07 ± 95.53	39.85 ± 3.25
Alkanes:						
Cadalin	101.34 ± 33.4	72.69 ± 20.95	ND	123.5 ± 5.13	186.11 ± 117.09	54.63 ± 4.06
trans-Calamenene	832.56 ± 114.54	508.43 ± 84.7	ND	822.76 ± 545.5	1301.58 ± 746.3	ND
(-)-g-Cadinene	ND	122.87 ± 20.35	ND	ND	1680.11 ± 620.89	ND
Ketones:						
Zonarene	ND	81.1 ± 13.23	ND	ND	ND	ND
1-(2,4,5-Trimethoxyphenyl)butan-1-one	ND	ND	ND	ND	ND	43.88 ± 0.98
Bicyclohexyliden-2-one	ND	74.24 ± 10.67	ND	ND	ND	ND
Phenols:						
Neointermedeol	ND	ND	ND	ND	ND	101.89 ± 47.65

ND indicates not detected.

Principal component analysis (PCA) is a technique that shows general variation between groups and variation within groups. In the results of PCA, the first principal component (PC1) explained 32.49% of the total variance. Different distributions indicated different maturity stages of the samples. The second principal component (PC2) explained 26.2% of the total variance (**Figure 4B**). In addition, significant differences between cultivars and periods were found.

Row standardized cluster analysis [values taken as log2 (value + 1)] was performed on the volatile compounds detected from jaboticaba, and the results are shown in the heat map (**Figure 4C**). It is clear that the types and contents of volatile compounds detected varied between periods and cultivars.

The composition of volatile compounds is the basis for the flavor provided by the fruit, consistent with previous studies in jaboticaba (Plagemann et al., 2012), and terpenes and alcohols were the predominant aromatic compounds in jaboticaba. However, the volatile compounds of jaboticaba is lower than that of grapes (Wu et al., 2020). There were differences in the aromatic composition of the three cultivars of jaboticaba. For ripe fruit, Sabara and Argentina had more abundant volatile compounds, which makes them more suitable for fresh consumption. In contrast, Fukuoka had a more homogeneous composition, as only six volatile compounds were detected, despite the fact that it detects the most volatile substances when unripe. Since the aromatic profile was limited, Fukuoka was not considered suitable for fresh consumption. As mentioned earlier, the fruit size of Fukuoka were larger, which also confirms its suitability for intensive processing.

## 4 Conclusion

In this study, it was found that generally, three jaboticaba cultivars berry were rich in nutrients, high in polyphenols content antioxidant capacity. However, there were significant differences in their nutrient content and volatile compounds. Sabara has a suitable acid-to-sugar ratio, superior flavor and slightly lower antioxidant capacity for fresh consumption. Argentina, although it does not taste as good as Sabara, has a high antioxidant capacity and is the richest in volatile compounds, making it suitable for development of dry products. The large size, juicy flesh and low sugar-acid content of Fukuoka also make it suitable for juice processing. The results could promote the development of the jaboticaba industry and broaden potential applications in the field of health products. Selective cultivation and processing of jaboticaba cultivars could lead to numerous health and economic benefits.

## Data availability statement

The original contributions presented in the study are included in the article/supplementary material. Further inquiries can be directed to the corresponding author.

## Author contributions

SX: conceptualization, methodology, validation, writing - original draft, and data curation. YP: methodology, validation, and writing - original draft. XC: methodology, validation, and data curation. QC: formal analysis and writing - original draft. GJ: conceptualization and writing - review and editing. MZ: conceptualization and writing - review and editing. LH: conceptualization, writing - original draft, writing - review and editing, methodology, and validation. All authors contributed to the article and approved the submitted version.
